# Weight and body image during pregnancy: a qualitative study of the experience of pregnant women, midwives and dietitians

**DOI:** 10.1080/17482631.2025.2608194

**Published:** 2025-12-25

**Authors:** Isabelle Carrard, Raphaël Hammer, Cindy Chevalley Gerber, Marielle Schmied

**Affiliations:** a Department of Nutrition and Dietetics, Geneva School of Health Sciences, HES-SO University of Applied Sciences and Arts Western Switzerland, Carouge, Switzerland; b HESAV School of Health Sciences - Vaud, HES-SO University of Applied Sciences and Arts Western Switzerland, Lausanne, Switzerland

**Keywords:** Body image, gestational weight gain, pregnancy, healthcare providers, women’s perceptions, perinatal care, qualitative research

## Abstract

**Purpose:**

Pregnancy is a time of rapid physical transformations. Medical and societal pressures regarding women’s weight and body image throughout pregnancy may increase body dissatisfaction, which can negatively affect psychological health and health behaviors. Yet healthcare providers (HCPs) often feel uncomfortable addressing the topic. This study explored women’s experiences of body changes during pregnancy, as well as the practices and challenges faced by midwives and dietitians in supporting them.

**Methods:**

A purposeful sample of 20 pregnant women (16−32 weeks of gestation) in Switzerland participated in face-to-face semi-structured interviews. In addition, four focus groups were conducted with six midwives and four dietitians. All narratives were transcribed verbatim and analyzed using thematic analysis.

**Results:**

Three themes were identified in pregnant women’s interviews: navigating body changes, managing the unmanageable, and experiencing lack of support around body image and weight gain. Women worried about weight gain, attempted to monitor their diet, and often felt unsupported by HCPs. Two themes were identified in HCP focus groups: reassuring and conveying information, and experiencing practical obstacles and societal challenges. HCPs acknowledged the sensitivity of the topic and described adopting a reassuring stance while conveying information and seeking ways to help women make peace with their bodies.

**Conclusions:**

Tailored support for body image during pregnancy is needed to promote maternal and fetal health. Midwives and dietitians are well placed to provide interdisciplinary consultations addressing gestational weight gain and body dissatisfaction. Training in positive body image could enhance their confidence in addressing these issues.

## 1 Introduction

Pregnancy is a time of profound physical transformations, characterised by rapid weight and body changes. Both excessive and insufficient gestational weight gain (GWG) are associated with numerous adverse maternal and foetal outcomes (Goldstein et al., 2017; Langley‐Evans et al., 2022). To promote healthy pregnancy weight trajectories, the U.S. Institute of Medicine (IOM) has established GWG thresholds based on pre-pregnancy body mass index (BMI; Institute of Medicine, 2009). However, a systematic review pooling data from studies conducted in the United States, Asia, and Europe reported that 47% of pregnant women exceeded these recommendations, while 23% gained less than advised (Goldstein et al., 2017). The IOM further recommends that healthcare providers (HCPs) communicate these guidelines to pregnant women according to their pre-pregnancy BMI (Institute of Medicine, 2009). They should also monitor and discuss weight progression throughout pregnancy, and provide tailored advice on nutrition and physical activity, which are key modifiable factors for supporting appropriate GWG.

While pregnancy may increase motivation to adopt healthier behaviours (Phelan, 2010), it also introduces multiple barriers to lifestyle changes, including physical symptoms, additional responsibilities, and elevated stress (Gagnon et al., 2023; Hill et al., 2017; Vanstone et al., 2017). For some, limited professional and family support may further hinder engagement in health-promoting behaviours (Gagnon et al., 2023; Hill et al., 2017; Vanstone et al., 2017). Beyond genetic predispositions and lifestyle behaviours, researchers have increasingly highlighted the role of maternal psychological factors in influencing GWG (Bergmeier et al., 2020). In particular, body dissatisfaction, together with depression and low social support, has been associated with excessive GWG (Hartley et al., 2015). Body dissatisfaction, which is defined as the difference between how a person perceives their own body image and how they think they should look, is very common among women in Western societies (Calogero et al., 2007). As a multidimensional construct, body image refers to the internal representation individuals form of their body, including perceptive, cognitive, affective, and behavioural aspects of body experience (Cash, 2004). This internal representation does not necessarily correspond with actual appearance or conformity to cultural beauty standards, making body dissatisfaction invisible from the outside. During periods of rapid body change, such as pregnancy, women must adapt to a new physical reality, leading to a reevaluation of their body image (Tiggemann, 2004). Prospective studies have shown that body dissatisfaction predicted both prenatal and postpartum depression (Riquin et al., 2019; Silveira et al., 2015). Body dissatisfaction has also been linked to shorter breastfeeding duration (Brown et al., 2015) and with postpartum weight retention (Phillips et al., 2014).

Systematic reviews of qualitative studies show that pregnant women often experience ambivalent feelings toward their bodies, encompassing both appreciation and discomfort (Hodgkinson et al., 2014; Watson et al., 2015). While weight gain is generally acknowledged as necessary and indicative of healthy foetal development, physical appearance often remains a significant concern. Many women also report a temporary shift in priorities: the body’s function may take precedence over its appearance, which can foster greater acceptance of physical changes (Clark et al., 2009; Watson et al., 2016). Nonetheless, cultural pressures persist even during pregnancy, with many women reporting feeling pressure to limit their weight gain and to quickly return to their pre-pregnancy shape after giving birth (Clark et al., 2009; Rodgers et al., 2024b).

Perinatal HCPs have a key role to play in helping women adjust their health behaviours during pregnancy and counselling them about weight and body changes. Pregnant women have reported receiving little information from HCPs (Gagnon et al., 2023) and described inconsistent messaging about GWG, which undermines the importance of the topic (Vanstone et al., 2017). Many women have expressed a desire for regular, constructive discussions about weight gain, along with tailored advice on nutrition and physical activity (Nikolopoulos et al., 2017). These findings align with reports from prenatal HCPs, who often lack confidence in addressing body image and GWG, citing insufficient knowledge and time constraints as key barriers (Plante et al., 2020). Additionally, HCPs may avoid the topic for fear of inducing shame, guilt, or reinforcing weight stigma (Christenson et al., 2018).

A deeper understanding of pregnant women’s experiences with their bodies, alongside the practices of perinatal HCPs, is essential to improve communication about GWG and body image during this critical life stage, thereby fostering health-promoting behaviours and maternal well-being. While most studies have examined women’s accounts and HCP perspectives separately, our aim was to place the two experiences in perspective. We chose to interview midwives and dietitians as HCPs, acknowledging that other perinatal HCPs could also have been included. These two groups were of particular interest. Midwives bring a holistic perspective on women’s health. Their position to discuss lifestyle advice and GWG is considered privileged, as they provide continuity of care that other HCPs often do not (Bahri Khomami et al., 2021). Dietitians, for their part, are not systematically involved in pregnancy care, although their specialized expertise in nutrition has been shown to improve certain birth outcomes (Hanifi et al., 2023; Mitchell et al., 2017). While the attitudes of midwives toward gestational weight gain have been well studied (Raju et al., 2023), to our knowledge, few studies have explored the attitudes of dietitians on this topic. Their perspectives are both highly relevant and complementary to each other, as well as to the gynaecologist’s medical approach in managing weight and body image during pregnancy. This study, therefore, sought to explore women’s experiences of body changes during pregnancy and to examine the attitudes and practices of midwives and dietitians, as well as the challenges they face in addressing weight and body image in perinatal care. The research questions were formalised as follows: (1) How do women experience the body changes that occur during pregnancy? and (2) What are the attitudes, practices, and challenges of midwives and dietitians in addressing weight and body image during pregnancy?

## 2 Methods

### 2.1 Research design

This qualitative study was informed by critical realism and contextualism (Braun & Clarke, 2006) and drew on data from a broader project exploring pregnant women’s food-related experiences and HCPs’ perspectives. It involved two steps: semi-structured face-to-face interviews with pregnant women, followed by focus group discussions with midwives and dietitians, the latter being informed by insights from the interviews. The Consolidated criteria for reporting qualitative studies (COREQ; Tong et al., 2007) were used to report the study (Appendix 1).

### 2.2 Research team

The research team brought together members with complementary knowledge and expertise. IC, a professor with a PhD in psychology who identifies as a woman, conducts research on eating behaviour and body image across the lifespan, working with a cognitive-behavioural framework. RH, a professor with a PhD in sociology who identifies as a man; his research focuses on women’s lived experiences of health risk during pregnancy. CCG, a young researcher with a MA in social sciences who identifies as a woman, was the project’s research fellow; she had prior experience in qualitative research when working on her master’s thesis on “The first 1000 Days”, which focuses on the period from conception to two years of life. MS, a midwife and lecturer who identifies as a woman, with an MSc in Global Health Policy, and specialized in health promotion during pregnancy.

### 2.3 Ethical consideration

The study adhered to the ethical guidelines of the Swiss Academy of Humanities and Social Sciences, following the Declaration of Helsinki. The local ethics committee determined that the study did not fall within the scope of the Swiss Federal Human Research Act, because no health data was collected (Req-2023-00773). Therefore, a formal ethics review was not required. All participants received an information sheet detailing the objectives of the study, their right to withdraw at any time and the confidentiality of collected data. Written informed consent was obtained from pregnant women prior to their interviews, and oral consent from HCPs, as focus groups were conducted online. To ensure anonymity, all participants’ names were replaced with pseudonyms in the interview and focus group transcripts. These pseudonyms are used when presenting quotations in the results section.

### 2.4 Participants and recruitment

The study took place in the French-speaking part of Switzerland. All participants received a 20 Swiss franc (around 20 USD or 20 EUR) gift card as a token of thanks for their time.

#### 2.4.1 Pregnant women

A purposeful sample of 20 pregnant women was recruited through snowball sampling, flyers, announcements on the researchers’ institution's websites and social media, and information sent to prenatal centres and birth preparation classes. The inclusion criteria included being between the 16^th^ and 32^nd^ weeks of gestation, being over 18 years of age, having no medical complications, and not having an active eating disorder. BMI was not considered a criterion.

#### 2.4.2 Healthcare professionals

Six midwives and four dietitians were recruited to participate in the focus groups. The inclusion criterion was to have experience in providing support during pregnancy. Two members of the research team who work in these professional fields used a snowball method to recruit participants. This method was deemed relevant for obtaining samples of professionals with the desired experience, particularly dietitians, as there are few practitioners specialized in pregnancy in the recruitment region.

According to Braun and Clarke (2006) recommendations for a medium-sized exploratory study, sample sizes of 20 pregnant women for interviews and of 4 focus groups for HCPs were deemed sufficient to provide rich data for thematic analysis. Data saturation was not sought (Braun & Clarke, 2021).

### 2.5 Data collection

#### 2.5.1 Pregnant women

Individual interviews were conducted by CCG between August 2023 and January 2024, in a quiet place convenient for the participants. Each interview lasted between 60 and 90 minutes. The interviews were audio-recorded and transcribed verbatim. A student hired for the task transcribed eight of the 20 interviews, and the research team transcribed the rest. A semi-structured interview guide was developed. The young researcher who conducted the interviews first pilot tested it with a pregnant acquaintance who was not part of the study sample. The first interviews of the study were also used to further adjust the guide, improving its clarity and ease of use. The interview guide comprised open-ended questions addressing changes in eating habits, beliefs about the ideal pregnancy diet, nutrition-related information-seeking behaviours, perceived information needs, and challenges encountered in dietary changes. The development of the interview questions was guided by the research questions, insights from the literature, and the team’s expertise regarding body image and managing weight-related issues during pregnancy. The specific questions relevant to the present analysis are presented in Table 1.

**Table 1.** Interviews and focus group guides.

**Selected questions from the interviews with pregnant women**
What do you think of the saying “you should eat for two” when you're pregnant?
Some women do not gain enough weight during pregnancy, while others are at risk of gaining too much. Is weight gain a concern for you?
Some women feel more fulfilled in their bodies when pregnant, while others find it more difficult to cope with body changes. Where do you stand?
Does weight gain or your body image affect how you eat during your pregnancy?
Are you able to discuss your concerns with a healthcare professional or with people around you?
Would you have liked to receive more information on the topic of weight gain during pregnancy?
**Selected questions from the focus groups with healthcare professionals**
What information should be shared about weight gain during pregnancy?
In your opinion, are women sufficiently informed that weight gain is both normal and necessary?
If you weigh the patient or have access to their weight data, how do you address it?
Weight gain during pregnancy and postpartum weight loss are common concerns among pregnant women. Is this something you discuss during consultations?
What obstacles or challenges do you face in your professional practice when discussing diet or weight?

#### 2.5.2 Healthcare professionals

Four focus groups lasting between 87 and 101 minutes were conducted via videoconference between April and May 2024: two with midwives (*n* = 4 and *n* = 2, respectively) and two with dietitians (*n* = 2 in each group). The midwives' focus groups were facilitated respectively by CCG and MS, and CCG and RH; the dietitians’ focus groups were facilitated by CCG and IC. The discussion guide included themes that we identified as critical during the interviews. One such theme was the importance of keeping weight gain low during pregnancy. We included interview excerpts about the advice women expected to receive regarding food and their concerns about weight gain. The goal was to spark discussions among HCPs about these narratives. Additionally, the discussion guide focused on professional experience with pregnant women, key messages to convey regarding diet and eating behaviours, perceptions about the importance of addressing weight gain and postpartum body image, as well as perceived challenges and barriers in providing information on these topics. The focus group discussion guide was not pilot tested, and no modifications were deemed necessary following the first focus groups. The questions specifically contributing to the present analysis are listed in Table 1. All sessions were video-recorded and transcribed verbatim by the research team.

### 2.6 Data analysis

The data were analysed using the reflexive thematic analysis approach (Braun & Clarke, 2022). This method was selected for its theoretical flexibility, allowing the research team to integrate and articulate their multiple disciplinary perspectives in the analysis and interpretation. The analysis involved six iterative steps: (1) familiarisation with the data set: all four research team members read the transcripts and shared preliminary insights; (2) systematic data coding: the segments of data specifically related to weight and body image during pregnancy were coded inductively by IC, using MAXQDA software (VERBI Software, 2025); (3) generating initial themes from the coded data, and (4) developing and reviewing themes: IC presented initial themes for discussion and review in meetings with the research team to ensure coherence and relevance; (5) refining, defining, and naming themes: the research team agreed on a final presentation of the themes; (6) writing: IC drafted the first analytical narrative, which was then revised by the research team.

### 2.7 Trustworthiness

Credibility was ensured through regular team debriefings during data collection, which provided opportunities to reflect on preliminary insights, adapt the semi-structured interview guide accordingly, and develop guidance for the focus groups. The involvement of the research team members at various stages of the research process also contributed to investigator triangulation, particularly during the coding and theme development phases, ensuring diverse perspectives in data interpretation. We also paid close attention to negative cases in the analytical process. Feedback from participants was not sought regarding the findings.

## 3 Results

### 3.1 Pregnant women - interviews

Gestational age ranged from 17 to 35 weeks (mean = 24.8 weeks, standard deviation = 6.5), with most participants (*n* = 14) being in their second trimester and six in their third. Participants ranged in age from 22 to 39 (mean age = 31.8, standard deviation = 4.9). Sixteen women were expecting their first child, and four were expecting their second. All the participants were receiving obstetric care from a gynaecologist, with three also followed jointly by a midwife. Sixteen had completed tertiary education, three had upper-secondary qualifications, and one was completing a bachelor’s degree. The majority of participants were Swiss or bi-national (*n* = 13), and most were in a stable romantic relationship (*n* = 18). Half of the participants (*n* = 10) worked in the health sector (e.g., nurse, medical assistant).

The thematic analysis of the interviews conducted with the 20 pregnant women led us to identify three core themes—Navigating body changes, Managing the unmanageable, Experiencing lack of support around body image and weight gain—and eight sub-themes (Table 2).

**Table 2.** Themes and sub-themes generated from the interviews with pregnant women.

Themes	Sub-themes	Example of quotes
Navigating body changes	*Being worried about body changes*	*Let's say it's becoming an issue anyway. worrying about* *“okay, how much weight have I gained?” But it’s more a fear of gaining too much [weight] than not enough. (Ivana, 19 WG)*
	*Adopting a pragmatic attitude: It’s only temporary*	*I'm okay with weighing this much for now. In a few months, I probably won't be anymore. (Julie, 35 WG)*
	*Loving one’s pregnant body*	*I feel great about my body. I've gained quite a bit of weight, but I’m feeling good. Apparently, my belly is fairly big compared to others at my stage, but I like its roundness. (Patricia, 35 WG)*
	*Worrying for the postpartum period*	*To be honest, I'm a bit apprehensive about the postpartum period. I'm a little worried that I won't get my original body back. I'm afraid of gaining too much weight and then having trouble losing it. (Dina, 18 WG)*
Managing the unmanageable	*Efforts to eat healthily and limit weight gain*	*I'm trying to be a little more careful about what I eat so that I don't gain too much weight. (Nina, 27 WG)*
	*Relying on internal sensations*	*So it was quite natural. […] I told myself, “Listen to yourself a little more. When you feel like eating, eat. When you don't, don't.” And it just happened naturally. (Patricia, 35 WG)*
Experiencing lack of support around body image and weight gain	*Absence of dialogue about weight gain.*	*In fact, not even my gynaecologist mentioned it to me. She didn't say, “On average, you should gain this many pounds. If you gain more than that, it's not good, and if you gain less than that, it's not good either.” (Béatrice, 31 WG)*
	*Ambiguous or inappropriate comments*	*My gynaecologist […] told me, “Before the 18th week, there's no point in gaining weight. It's not the baby gaining weight, it's you.” (Thaïs, 17.5 WG)*

Note: WG: weeks of gestation.

#### 3.1.1 Navigating body changes

*Being worried about body changes.* Experiencing rapid body changes was a source of psychological discomfort for most pregnant women in our study. They expressed a sense of being out of control, with uncertainty about how much weight they would gain and what the lasting impact on their body shape and skin would be. The expression of these concerns mainly varied depending on the weight gained. When weight gain was still minimal, women were mostly concerned with how their body image would evolve, as said by Dina (18 weeks of gestation; WG): “*I'm still in the early stages, so I don't really know what's going to happen afterwards, but it's true that I don't necessarily want to put on too much weight*.”

In contrast, some women who had already experienced significant weight gain according to the medical standards described strong aversions to their body changes. For example, Wanda (21 WG), felt powerless in front of her body’s changes: “*I hate it, I see myself in the mirror, I want to run away, I think I look awful*.”

The concern women described did not seem to be related to concerns about the body before pregnancy. Those who claimed to have a natural tendency to gain weight worried that pregnancy might exacerbate this trend. At the same time, women who had always been thin and remained within the advised pregnancy weight range also expressed distress about their changing bodies. They struggled with feeling superficial for caring about their appearance when the important thing was that the pregnancy was going well:

*I realize, when I say it out loud, that it's a bit superficial, that if the child is well, that I'm well too, that's really the most important thing, and I know it, but still, I know that, for me, appearance is also important, and these are also questions I have. (Romane, 17 WG).*

Not all body parts were “allowed” to grow. For some women, an appreciated change was a visible belly, and for some, larger breasts. These changes were seen as healthy and functional, showing that the pregnancy was progressing well. It also showed the world that the woman was pregnant and not just fat. One of the four participants who were expecting a second child reported having had the kind of pregnancy every woman would dream of, in terms of weight gain and how it was distributed on her body, illustrating what the ideal pregnant body was supposed to be:

*I think I've got everything a woman could wish for, I take exactly what I need and I lose it very quickly [...] with my first pregnancy I put on 10 kilos and it was only visible on my stomach, people looked at me from behind at 8 and a half months pregnant, they couldn't tell I was pregnant, and after 3 months I was back to my normal weight. (Umi, 20 WG).*

Supportive comments from partners or friends could be protective. Positive comments were particularly appreciated, such as noting that the weight gain was not obvious, or reassuring them that there was no need to worry because a baby was on the way. Conversely, participants may also encounter critical or judgmental comments that were difficult to deal with:

*I'm lucky to have my partner, who's very caring and reassures me, and that's good. But then I've had friends come up to me not long ago and say “Maya, you've put on weight”, or my family saying “you should do some sport because...” or “stop eating because you've put on too much weight”. (Maya, 19 WG).*

*Adopting a pragmatic attitude: It’s only temporary.* Instead of expressing concerns about weight changes, some participants adopted a more pragmatic approach toward body changes and weight gain. Although not thrilled by these transformations, they acknowledged them as expected. They recognized their transitory nature and focused on positive signs that the pregnancy was progressing well. This form of cognitive acceptance also reflected a shift in priorities. For instance, Julie (35 WG), who had previously feared these changes and considered herself superficial for doing so, was surprised at how easily she accepted them during pregnancy: “*I accept this weight quite easily because I say to myself, well, if I hadn't taken them, the baby wouldn't be able to... well, it needs this, actually*.”

Another form of rationalisation involved focusing on the small amount of weight gained and the fact that most of it was concentrated in the belly area. For example, Gaëlle (25 WG) who lost weight at the start of her pregnancy: “*So in 6 and a half months, I've only put on 3 kilos. Well, that's almost nothing, so I figure that for the moment, I'm living it pretty well.”*

Finally, some participants focused on functionality rather than on appearance, as their bodies became painful and less mobile. They expressed a desire to regain their physical abilities:

*In the second trimester, I was in better shape, so I felt pretty good, pretty fulfilled in the sense that my belly was starting to round out, but I could still go for walks, do my activities, etc. [...] and now, on the other hand, I have to admit that I... I feel a bit rented, like I'm taking someone in (laughs), and that sometimes handicaps me from doing things. (Ophélie, 34 WG).*

*Loving one’s pregnant body.* Three women described a deep love and admiration for their pregnant bodies. They found the changes “magical”, a term used by Charlotte (18 WG) who commented: “*Well, it's quite magical actually, […] to see this body being transformed without you doing anything about it*.”

Two of these women had conceived after several unsuccessful attempts using assisted reproductive technology. Their current pregnancies were experienced as particularly precious and long-awaited, which likely influenced their perceptions. For example, despite significant weight gain, Patricia (35 WG) had a positive perception of the body transformations: “*I like being pregnant. I like this thing, I like my belly. I like this belly*.”

The third woman, Béatrice (31 WG), expressed her genuine preference for her pregnant body, noting that her clothes fit better. She was deeply connected to the idea that her body was creating life: “*I like the shape it's in, with a baby in it, and I think the fact that I can see the whole side too, that's really what life is like*”.

*Worrying for the postpartum period.* Regardless of their attitude toward weight gain while pregnant, participants expressed worries about the postpartum period. Their concerns included anticipating how their bodies would look after childbirth, how to lose the weight gained during pregnancy, and how to return to their pre-pregnancy bodies:

*I can't imagine myself ten kilos heavier. But I tell myself it's just - during pregnancy it's normal, let's say it's the aftermath that scares me, because I want to be able to go back to the way I was before. (Nina, 27 WG).*

While most participants acknowledged that it would take time to regain their pre-pregnancy bodies, some were eager to resume physical activity and control their diet to speed up the process:

*My goals are to get back into my pre-pregnancy clothes as quickly as possible, lose weight as soon as my doctor gives me the OK, and get back into sports. I want to lose the pregnancy weight as quickly as possible. (Fanny, 33 WG).*

The idea of “just taking the necessary weight” for the baby, no more, was commonly verbalized. For example, Charlotte, who lived with being overweight and had previously lost weight in preparation for pregnancy, hoped to avoid weight loss efforts after birth:

*After all, I think we all have this ideal of saying to ourselves, well, if I put on just the right amount of weight to have a baby, and once I've given birth there's no more weight to lose, that's it, if it could be between 7 and 10 kilos, well, that would be perfect. (Charlotte, 18 WG).*

Only one participant resisted the cultural injunction to “get her body back”. For Béatrice, the irreversible physical traces of pregnancy were signs of lived experience. Health was the one thing that mattered:

*As for the phrase “you have to get your pre-pregnancy body back”, well, you haven't lost your body, girl, I mean... and yes, there will be marks because it's obviously been through a crazy experience, so whether you've got a few more stretch marks or it's a bit softer, well, it just shows what you've been through. (Béatrice, 31 WG).*

#### 3.1.2 Managing the unmanageable

*Efforts to eat healthily and limit weight gain.* Many participants reported monitoring their diet to manage weight gain. Only a few participants mentioned physical activity, and never as a main strategy. The main strategy was to eat a healthy diet, most of the time without more specific details about what a healthy diet entailed:

*There's also the importance of eating healthily and making sure that it's good for our bodies too, so that we don't... well, maybe it's a bit futile, but that we don't put on too much weight either. (Julie, 35 WG).*

When asked for details, participants described strategies that included reducing their sugar intake, not giving in to their hunger or cravings, and avoiding foods that they considered to be unhealthy. These strategies were derived from general dietary principles, without personalisation. Striving to adhere to such dietary practices was seen as a preventive strategy to limit weight gain and facilitate postpartum recovery:

*I don't really indulge even during pregnancy, I don't eat too much salt, too much sugar or too much fat, I try to eat as healthily as possible. To be healthy in my mind too, because I feel very guilty about eating things, salty things, desserts, because I know it won’t help me lose weight afterwards. (Fanny, 33 WG).*

Most participants mentioned that the social environment was not very supportive. They reported that friends and family encouraged them to eat whatever they wanted “for once” during this period of their lives and downplayed their concerns about weight gain:

*My family and friends tend to say to me, "Go ahead and eat it, if you feel like it, don't hold back or anything." Even my in-laws, sometimes I avoid saying that I want something because if I say it, they'll want to give it to me and then want me to eat it, when I know that I shouldn't overindulge in sweets.” (Hélène, 28 WG).*

Two participants reported that they were unable to adopt healthy eating habits, even after the early symptoms of pregnancy had disappeared. One, experiencing her second pregnancy, was affected by pregnancy-induced hyperemesis that caused a deep aversion to food. The second participant was a midwife who was convinced by her studies that this period of life was ideal for implementing healthy dietary principles. However, she had found herself unable to apply what she had learned. She could not explain why, which triggered self-blame and self-questioning:

*I had quite a few big - I wouldn't say “big principles”, but I told myself [...]: “I'm going to be super careful, I'm going to be super strict, I'm going to eat almost no sugar, it'll be really good for the baby and everything”. So, I didn't do that at all, I don't even know if I succeeded for two weeks, well really... I don't even know if I really tried in the end, I'm a bit ashamed, because I say to myself "did you really try? (Salomé, 27 WG).*

*Relying on internal sensations.* A small group of participants found it easy to rely on their internal sensations of hunger and satiety. These women described eating as a relatively intuitive process based on body cues rather than external norms. They reported that their bodies naturally signaled when they had eaten enough, such as Maya (19 WG), who reported: “*I know my body tells me when it's too much, it tells me and I don't eat more, I can't”.* Also, these participants did not experience strong cravings.

This trust in body cues was encouraged by HCPs and valued by the women. However, concerns remained about the potential impact on weight gain, particularly when hunger was perceived as stronger than before. Romane (17 WG), who was convinced that relying on her internal sensations was a better way of nourishing herself and her baby, rather than relying on external standards, said: “*So I really listen to my hunger. Sometimes it's a bit confusing because I feel like I'm eating all day long. But I tell myself that my body knows what it needs.”*

Some participants who tried to follow their internal sensations, as recommended by HCPs, found them to be unreliable. For example, Nina (27 WG) said that identifying hunger and satiety had never been easy, and that her HCP’s recommendation to eat her fill was not helpful: “*I don't think it's easy. Especially when you have cravings, sometimes you feel you're really hungry when it's actually just a craving”.*

#### 3.1.3 Experiencing lack of support around body image and weight gain

*Absence of dialogue about weight gain.* Many participants reported a lack of support regarding weight gain from their gynaecologist or other HCPs during pregnancy. The participants’ body image was never a topic of discussion. Participants were surprised that expected weight gain was not consistently addressed during medical consultations. For example, Charlotte (18 WG) was never weighed during her appointments: “*I ask myself about weight gain alone because my gynaecologist never weighed me or talked to me about weight gained during pregnancy.”*

Other participants were weighed routinely, but without accompanying feedback or discussion. Several participants interpreted this silence positively, assuming that the HCP would address the issue if weight gain was problematic. They may even appreciate the lack of “pressure”. However, others expressed a desire to have the opportunity to discuss weight gain with their gynaecologist or midwife:

*They weigh you and note it down, but they don't comment on whether the weight is good or bad. Even during my first trimester, when I lost weight, they didn't comment on it. I was the one who asked the questions*: *“Is that normal?” I think they could talk a little more. (Nina, 27 WG).*

Participants described midwives as more accessible than gynaecologists, but still hesitated to initiate conversations about weight, perceiving it as a “secondary issue”. In the absence of professional guidance, several women searched for information on their own to estimate the “right amount” of weight gain:

*Since my gynaecologist kept telling me to “be careful” at every appointment, I wondered why. That’s why I did research on weight too. I wondered if she was telling me that because I was gaining too much weight and if that was bad for me or the baby or whatever. It did worry me a little, so I looked up some information and saw that women can gain more or less weight depending on their initial weight. (Hélène, 28 WG).*

*Ambiguous or inappropriate comments.* While weight was occasionally addressed in the HCP consultations, the messages were often unclear or anxiety-provoking. Other comments were awkward or poorly phrased, such as a midwife’s surprised reaction to Ophélie’s (34 WG) weight gain: “*At my last appointment with the midwife, I saw that I'd gained 14 kilos since the beginning, so that's a lot, and so she said to me “Yikes! what have you done?”*

Although these comments were sometimes interpreted as well-meaning rather than guilt-inducing, they were not considered helpful because they did not provide women with concrete lifestyle advice. One participant, Thaïs (17.5 WG), described feeling stressed during a previous pregnancy due to her gynaecologist’s comments: “*And it's true that I think the gynaecologist contributed to the fact that it was stressing me out, because he would sometimes say to me, “ah but it's still a bit too much.”*

### 3.2 Health care professionals—focus groups

The six midwives who participated in the focus groups had between five and 29 years (mean = 14.7 years, standard deviation = 9.8) of experience in the field. They were engaged in providing care during pregnancy and the postpartum period, either in group or individual format, within institutions or private practices. Their patients came from diverse backgrounds, including low-income or migrant populations. The four dietitians had between two and 14 years (mean = 8.8, standard deviation = 5.0) of experience working with pregnant women. They were involved in institutional services dedicated to maternity care, private practice, or offered free nutrition classes to groups of pregnant women through associations.

We generated two core themes—Reassuring and conveying information, Experiencing practical obstacles and societal challenges—from the focus group discussions (Table 3).

**Table 3.** Themes generated from the focus groups with midwives and dietitians.

Themes	Examples of quotes
Reassuring and conveying information	*[…] Then, work on limiting any feelings of guilt or concern about body image... but weight issues affect many aspects of women’s lives and are complicated. (Line, midwife)* *If we just tell them “You have to do this, you mustn't do that,” they find it difficult to figure it out for themselves. I think starting with what they do and then applying the filter “This is great, this is something you should avoid,” helps them more. (Sylvie, dietitian)*
Experiencing practical obstacles and societal challenges	*It remains a minority [of patients treated by dietitians], and yet it is still a very important area. This is also highlighted by the WHO [...] saying that care must be taken during pregnancy and weight gain. But yeah, if you look at the figures, very few patients are treated by dietitians. (Claudine, dietitian).* *There are so many moms who are already under pressure their whole lives about their weight and body image. (Adrienne, midwife)*

#### 3.2.1 Reassuring and conveying information

All HCPs agreed on the sensitivity of the topics of weight and body image and the importance of alleviating anxiety in pregnant women. Weight gain was identified as a primary concern for pregnant women. They valued a reassuring and nonjudgmental approach to avoid exacerbating the guilt and anxiety experienced by pregnant women. They emphasised that excessive pressure was more stressful than helpful and could undermine the therapeutic relationship:

*I've also noticed that they're already under so much pressure that if you tell them “that's the wrong thing to do”, it puts even more pressure on them, and that also breaks the bond and adds to the stress [...]. So it's true that I'm always careful not to judge or appear judgmental. (Yolanda, dietitian).*

However, professionals did not avoid conveying important and clear health information. Finding the right balance between providing information and maintaining emotional stability was considered delicate and debated among HCPs:

*As you quite rightly said Adrienne [midwife participant], the aim really isn't to make them feel guilty, but I still think that for the well-being of their baby and for them too, by explaining things clearly as you said Line [midwife participant], if they gain 15 kilos it's not 15 kilos of fat that they're going to gain, of course not, but I think it's important to say that […] all the weight they put on is also [negative] for them, for their body, their heart, their joints, […], I think that by nuancing things a little in this way, we can convey the message without making them feel guilty and jump on the scales every morning. (Maeva, midwife).*

HCPs deemed it essential to personalise the advice they gave to pregnant women. When they suspected that a woman might be struggling with her weight or body image, whether due to being overly controlling, being unaware of recommendations, or lacking control over her dietary intake, they would take time to investigate and understand her specific situation. This helped tailor their advice to each woman’s needs, level of knowledge and emotional state. For example, Line, a midwife, would ask: “*What's behind this impression that she needs this control [of weight]? I think there's a real need to investigate and go further with her in reflecting on what weight gain is.”*

HCPs might explain the recommended weight gain range when they thought the woman had not yet been informed. However, this practice was not systematic, since it could put too much pressure on the pregnant woman. The information was presented in a flexible manner, emphasising the weight gain range and the fact that each case is different. Occasionally, they might explain the consequences of excess weight gain for the foetus and the woman, in order to give the information but with extra care to avoid provoking anxiety. As noted by Sylvie, a dietitian, HCPs deemed it important to normalise weight gain and explain why it was essential: “*Most importantly, we want to validate that it's normal to gain weight during pregnancy, as some women are worried and want to avoid it”*.

Focus group participants agreed on the importance of ensuring that pregnant women’s diets covered their nutritional needs, and of strictly discouraging restrictive diets, as underscored by Claudine, a dietitian: “*We also want to avoid these people falling into dietary restraint or restrictive dietary practices. That's why we support them. We absolutely don't want that to happen”*.

#### 3.2.2 Experiencing practical obstacles and societal challenges

HCPs pointed to concrete barriers in delivering tailored care to pregnant women. Although they emphasised the importance of adapting advice to each woman’s emotional state and knowledge level, they noted that working exclusively in group settings made this difficult. However, group formats could also be beneficial, allowing pregnant women to reassure each other and normalize their experience when they were struggling and felt that they had not reached the perfect standards they were aiming for:

*I very often have ladies who say “I'd like to do it [dietary changes] but I can't”, [...] and then typically the fact of being able to share with other moms and realize that they're not the only ones who can't do it, because it's more the rule than the exception. (Sylvie, dietitian).*

Professionals also regretted being involved too late in the pregnancy journey, especially in the case of dietitians, who were often consulted only once complications had emerged. Similarly, midwives were not always systematically included in pregnancy care. Dietitians and midwives claimed to have the time and expertise that could complement that of gynaecologists. They all called for an interdisciplinary initial consultation early in pregnancy to lay the groundwork for sensitive topics such as weight and body image:

*I think that each professional clearly has a role to play in contributing to our knowledge and supporting mothers during pregnancy and breastfeeding. I also believe that a combined consultation [...] to take time to discuss ... diet, weight gain, and physical activity, I think it's a really good addition to the medical side of pregnancy monitoring. (Emiliana, midwife).*

The pervasive societal pressure on women regarding weight and body image was mentioned as a problematic issue at multiple levels. HCPs did not always feel comfortable discussing body image or weight gain because some women were deeply concerned about the topics. However, HCPs themselves may also be worried about the topic, which complicates how they address it. For example, Adrienne, a midwife, has struggled with her own weight: “*I admit that for me it's a hypersensitive subject, but I think it's also due to my background, because I've struggled with my weight all my life”.*

HCPs observed that pregnant women felt strong pressure to quickly return to their pre-pregnancy bodies after childbirth. This added to women’s responsibilities of being a good mother and taking care of their newborn. Professionals who met with new mothers in groups reported frequent appearance comparisons among them regarding weight loss and described how social media played a detrimental role in shaping unrealistic expectations:

*Lots of moms refuse sweet things, saying “no, I'm on a diet”, and then a lot of “how much have you gained?”, “where are you with your weight loss?”, and all that, and a lot of guilt “ah she's already lost 4 kg”. (Dana, midwife).*

In response to growing concerns about postpartum weight, associations and institutions where the HCP participants worked launched new group sessions, which achieved notable success. These sessions were attended by a wide variety of women, regardless of the amount of weight they had gained during pregnancy.

*Even if [weight gain] isn't excessive, in fact, if it's not discussed with caregivers or others, it worries moms, and they're already anticipating weight loss, to such an extent that we've created a new course: “postpartum nutrition”, to talk about weight and weight loss. Most moms who come are not even moms who've gained too much weight but who want to lose it fairly quickly or they have an image of themselves that's not… they want to change, they don't feel good in their bodies (Yolanda, dietitian).*

To counter the misleading messages spread by social media about what a new mother “should” look like, HCPs emphasised the variety of postpartum experiences and the average timeline of about one year for achieving a stable weight. They also encouraged women to value self-care practices, such as looking in the mirror, massaging their belly, or observing their scars, as a meaningful step toward acceptance. In some cases, these conversations also opened the door to a deeper reflection on their new role and new identity:

*But there's still this concern, which isn't always about numbers but about the idea of going back to the body as it was before, and so I try to discuss with them what their body was like before, what has changed in their life, and why it's no longer the body it was before, and to move more towards acceptance of a mom's body and a mom's life (Adrienne, midwife).*

## 4 Discussion

Consistent with previous studies, pregnant women’s representations were shaped by societal expectations about how their bodies should appear as “pregnant” rather than “fat” (Hodgkinson et al., 2014; Watson et al., 2015). They described pregnancy as a time of heightened anxiety and anticipation, with varying levels of concern about weight gain and postpartum body changes. Some disliked their pregnant bodies and felt anxious about regaining their pre-pregnancy body shape, while others, more attuned to bodily function and the creation of life, felt unbothered or even appreciative of the transformations. These findings align with previous research highlighting a continuum of embodied experiences during pregnancy and the postpartum period (Hodgkinson et al., 2014; Rodgers et al., 2024a).

In parallel, the focus groups with midwives and dietitians highlighted the delicate balance between providing information and maintaining a supportive therapeutic relationship. Consistent with prior literature (Plante et al., 2020; Raju et al., 2023), the HCPs in our study acknowledged the sensitivity and importance of addressing weight gain and body image during pregnancy, but feared that raising these issues too directly might make women feel blamed, especially if they became involved later in pregnancy.

Taken together, the women’s interviews and the focus groups with HCPs, including dietitians, who are rarely investigated in this context, reflected patterns already described in the literature. Our study is the first to document these dynamics in Switzerland, highlighting the scope of weight-related issues and body image concerns during pregnancy in Western societies.

Contrary to earlier research, none of the participants in the women’s interviews reported feeling liberated from monitoring their weight during pregnancy or appreciating being allowed to gain weight (Nash, 2012). This trend highlights the increasing pressure surrounding the control of body shape and weight in Western societies, including pregnant women’s bodies. Furthermore, the concern about GWG appeared to be unrelated to the body image concerns women reported having before pregnancy. However, this finding must be tempered by the results of a quantitative study that showed a small longitudinal association between shape and weight concerns during adolescence and poorer body image in pregnancy and postpartum (Norton et al., 2024). It would nevertheless underscore the need to address body image with all women, regardless of body size.

While only one woman explicitly rejected the dominant discourse promoting rapid return to pre-pregnancy shape, most adhered to it. Sociocultural theories remain highly relevant in the peripartum context, explaining how media, family, and peers transmit beauty ideals, fostering internalisation, appearance comparison, and body dissatisfaction (Lovering et al., 2018; Rodgers et al., 2024b; Thompson et al., 1999). Social media further amplifies the dissemination of unrealistic images, which reinforce expectations of the idealised pregnancy body and lifestyle, and intensify body dissatisfaction among women (Rosenbaum et al., 2024). Pregnant participants also described receiving mixed messages from family and friends. They were encouraged to indulge in food but criticised for excessive weight gain. This illustrates the paradox of societal expectations around how women are supposed to behave during pregnancy.

These findings highlight the critical role HCPs could play in addressing body image concerns and promoting supportive dialogue during pregnancy (Nikolopoulos et al., 2017). Yet, most women reported that this topic was rarely, if ever, discussed during their medical consultations, or inadequately addressed, a gap also noted in previous studies (Nikolopoulos et al., 2017; Vanstone et al., 2017). The accounts of women in the present study suggest that HCPs did not consistently follow the IOM’s guidelines, which recommend informing pregnant women about GWG, helping them monitor their weight throughout pregnancy with regular weighing, dialogue, and advice about both diet and physical activity, including referral to relevant professionals when necessary (Institute of Medicine, 2009). Most women reported that they had not received GWG recommendations, and some who wanted such information had to search for it themselves. The narratives of the pregnant women in our sample did not align with the attitudes and practices described by the HCPs in the focus groups. Most women in our sample were followed primarily by their gynaecologist, which limits direct comparisons between the women’s and professionals’ narratives. Nonetheless, midwives and dietitians described attitudes and practices that closely aligned with what many women said they would appreciate: sensitive yet clear discussions that acknowledge their concerns. Compared to gynaecologists, midwives and dietitians may have greater flexibility to devote time to these topics, highlighting the complementary nature of their roles in perinatal care.

Women seeking guidance about the expected weight gain, sometimes holding unrealistic expectations of minimal weight gain, may turn to the Internet. However, besides providing advice that is less effective than that of HCPs, online information may reinforce normative ideals and stereotypical representations of the “perfect” pregnancy (Mercado et al., 2017). This lack of discussion with their HCPs may contribute to the belief that pregnancy weight gain corresponds only to the baby’s weight and that less weight gain is always preferable. However, insufficient weight gain can have maternal and foetal consequences, just as excessive weight gain (Goldstein et al., 2017).

Moreover, not all women in our sample reported being weighed, and for those who underwent regular weighing, it was performed without commentary. The evidence, however, shows that weighing alone does not influence GWG (Fealy et al., 2017). Some participants found this silence reassuring, while others wished for open discussion of their body image concerns, echoing previous findings (Nikolopoulos et al., 2017). Pregnant women hesitated to raise the topic, fearing they would burden HCPs with issues perceived as secondary or futile. This hesitancy underscores the importance of HCPs proactively opening space for body image discussions, as women will not initiate them.

Finally, women had not received tailored advice regarding dietary adjustments, and no women mentioned receiving advice on physical activity. Tailored support is essential for dietary adjustments, as also underscored by the HCPs in our study. Women in our study tried to adopt a “healthy diet”, symbolized by external rules promoted by public health, which, while appropriate, are difficult to adopt without personalisation (Hanifi et al., 2023), leading some women to feel powerless. While some participants tried to follow their internal sensations, others found this impossible or distressing. Although intuitive eating, guided by hunger and satiety cues rather than external rules, has shown benefits for GWG (Paterson et al., 2019), promoting it without concrete guidance can generate confusion, anxiety, and feelings of failure. In our study, dietary advice was often vague, leading some participants to blame themselves for their inability to eat more healthily, reflecting the widespread belief that weight and eating behaviour are entirely under personal control (Reinka et al., 2021).

The focus group results aligned with the literature on HCPs’ attitudes, practices, and challenges related to addressing body image and weight gain during pregnancy (Plante et al., 2020; Raju et al., 2023). The HCPs in our study reported difficulties addressing the topic of body image and weight gain, fearing it could harm the therapeutic relationship. However, they described adopting a thoughtful and careful approach to these topics, without avoiding the delivery of necessary information. HCPs reported having developed specific practices to address weight and body image concerns, such as maintaining a non-judgmental stance, asking exploratory questions, and tailoring messages to provide practical guidance. Previous studies have encouraged such strategies (Nikolopoulos et al., 2017). Given the diversity of women’s profiles and experiences regarding body changes, individualised and flexible approaches are essential. Notably, some women with healthy pre-pregnancy weight felt reassured by minimal GWG, a perception requiring attention. HCPs may be less inclined to explore such issues in women with a healthy weight (Plante et al., 2020), despite the risk that societal thinness ideals may prompt excessive dietary restraint (Bergmeier et al., 2020).

HCPs reported encountering practical obstacles, such as late referrals, group settings or time constraints, barriers also reported in previous studies (Plante et al., 2020). All HCPs also observed the pervasive societal pressures on women’s bodies during and after pregnancy. They deplored the obligations women imposed on themselves to control their diet and to regain their pre-pregnancy body shape, in addition to the responsibility of being a good mother, as noted in the literature (Hodgkinson et al., 2014). According to the HCP’s experience, women expressed a growing discontent about their bodies, which led HCPs to propose postpartum groups on the topic. HCPs had also developed attitudes aimed at helping women make peace with their new maternal bodies and supporting them in this new stage of life. Without explicitly naming it, they promoted a positive body image to their patients, particularly the facet of body acceptance, which means accepting one’s unique features without feeling obliged to conform to unrealistic sociocultural beauty ideals (Tylka & Wood-Barcalow, 2015). Positive body image is a multidimensional concept that has been shown to be protective of physical health, psychological well-being, and healthy eating behaviours (Linardon et al., 2022). It can be summarized in terms of three dimensions: (a) appreciating the body’s appearance and function, (b) being aware of and attentive to the body’s experiences and needs, and (c) possessing a positive cognitive style for processing body-related messages in a self-protective way (Halliwell, 2015; Menzel & Levine, 2011). Becoming more familiar with the concept of positive body image might help HCPs formalise their support for pregnant women in a more comprehensive way and help pregnant women resist societal pressures during this vulnerable time.

### 4.1 Clinical implications

In this study, most women were primarily followed by gynaecologists, whose limited consultation time and medical focus left body image concerns and weight gain largely unaddressed. In contrast, midwives and dietitians demonstrated great sensitivity to the topic and described concrete practices for engaging with these issues. They also expressed interest in interdisciplinary consultations, highlighting the value of combining their complementary expertise.

Targeted training could help legitimise HCPs’ roles in addressing body image and weight concerns and enable them to engage more confidently in these sensitive discussions. HCPs should be encouraged to address the topic with women, who often have the impression that it is superficial to have such concerns. Moreover, they should explain the IOM recommendations, focusing not only on the prevention of excessive GWG but also on the importance of gaining sufficient physiological weight, which is necessary for a safe pregnancy. Tailoring advice to support women in adopting a healthy lifestyle is essential to achieve meaningful change.

Providing HCPs with core concepts from the positive body image framework would enable discipline-specific interventions grounded in a shared integrative perspective. Interdisciplinary perinatal consultations involving specialists in body image, nutrition, and psychosocial adjustment could offer women a safe space to discuss their concerns early in pregnancy. Such integrative care may help prevent or mitigate the adverse consequences of body dissatisfaction for maternal and child health (Bergmeier et al., 2020).

### 4.2 Strengths and limitations

A key strength of this study lies in its qualitative design, which enabled an in-depth exploration of pregnant women’s body image experiences as well as the practices and challenges faced by midwives and dietitians. The parallel accounts of women and HCPs, with the pregnant women’s interviews used to enrich the HCP focus groups, made it possible to place both experiences into perspective. Among the limitations of this study, the women’s sample was mainly composed of white, tertiary-educated, first-time mothers, half of whom worked in the health sector, factors that may have influenced their perspectives and their gynaecologists’ attitudes. Most women were primarily followed by their gynaecologists rather than by midwives or dietitians, which limited the extent to which we could identify convergences or divergences between samples in our analysis. Additionally, the small size of three of the HCP focus groups (two participants each) may have limited the depth of discussion, but may also have encouraged the expression of every participant.

## 5 Conclusion

Pregnant women reported a lack of discussion regarding GWG and their changing body image. Midwives and dietitians are well-positioned to address these concerns but may require additional resources to support women in developing a positive body image. Interdisciplinary consultations could help leverage their complementary expertise and offer pregnant women a supportive space to discuss these issues and to protect themselves from societal norms surrounding weight and body shape during pregnancy.

## Supplementary Material

Supplementary File COREQ SAMALI_BI_REV1.docx Supplementary File COREQ SAMALI_BI_REV1.docx

## Data Availability

The participants of this study did not give written consent for their data to be shared publicly, so due to the sensitive nature of the research supporting data is not available.
