# Supplementary material for: Weight and body image during pregnancy: a qualitative study of the experience of pregnant women, midwives and dietitians
Source: Int J Qual Stud Health Well-being. 2025 Dec 25;21(1):2608194. doi: 10.1080/17482631.2025.2608194 (PMC12777822; doi:10.1080/17482631.2025.2608194)
Supplement: Supplementary File COREQ SAMALI_BI_REV1.docx [file ZQHW_A_2608194_SM4169.docx]

**Table S1: COnsolidated criteria for REporting Qualitative research (COREQ) - 32-item checklist**

| **Item number** | | **Guide questions/Description** | **Reported on page number** |
| --- | --- | --- | --- |
| **Domain 1: Research team and reflexivity**  **Personal characteristics** | | | |
| 1 | Interviewer/facilitator | Which author/s conducted the interview or focus group? | Page 9 |
| 2 | Credentials | What were the researcher’s credentials? (e.g. PhD, MD) | Page 7 |
| 3 | Occupation | What was their occupation at the time of the study? | Page 9 |
| 4 | Gender | Was the researcher male or female? | Page 9 |
| 5 | Experience and training | What experience or training did the researcher have? | Page 7 |
| **Relationship with participantss** | | | |
| 6 | Relationship established | Was a relationship established prior to study commencement? | Page 9 |
| 7 | Participant knowledge of the interviewer | What did the participants know about the researcher (e.g. personal goals, reasons for doing the research)? | Page 8 |
| 8 | Interviewer characteristics | What characteristics were reported about the interviewer/facilitator (e.g. bias, assumptions, reasons and interests in the research topic)? | Page 7 |
| **Domain 2: Study design**  **Theoretical framework** | | | |
| 9 | Methodological orientation and Theory | What methodological orientation was stated to underpin the study (e.g. grounded theory, discourse analysis, ethnography, phenomenology, content analysis)? | Page 7 |
| **Participant selection** | | | |
| 10 | Sampling | How were participants selected (e.g. purposive, convenience, consecutive, snowball)? | Page 8 |
| 11 | Method of approach | How were participants approached (e.g. face-to-face, telephone, mail, email)? | Page 8 |
| 12 | Sample size | How many participants were in the study? | Pages 8 |
| 13 | Non-participation | How many people refused to participate or dropped out? Reasons? | No drop-out. |
| **Setting** | | | |
| 14 | Setting of data collection | Where was the data collected (e.g. home, clinic, workplace)? | Pages 9 |
| 15 | Presence of nonparticipants | Was anyone else present besides the participants and researchers? | No. |
| 16 | Description of sample | What are the important characteristics of the sample (e.g. demographic data, date)? | Pages 12; 23 |
| **Data collection** | | | |
| 17 | Interview guide | Were questions, prompts, guides provided by the authors? Was it pilot tested? | Pages 9-10, Table 1. |
| 18 | Repeat interviews | Were repeat interviews carried out? If yes, how many? | No. |
| 19 | Audio/visual recording | Did the research use audio or visual recording to collect the data? | Pages 9-10 |
| 20 | Field notes | Were field notes made during and/or after the interview or focus group? | Yes, after interviews and focus groups. |
| 21 | Duration | What was the duration of the interviews or focus group? | Page 9 |
| 22 | Data saturation | Was data saturation discussed? | Page 9 |
| 23 | Transcripts returned | Were transcripts returned to participants for comment and/or correction? | No. |
| **Domain 3: analysis and findings**  **Data analysis** | | | |
| 24 | Number of data coders | How many data coders coded the data? | Page 11 |
| 25 | Description of the coding tree | Did authors provide a description of the coding tree? | No. |
| 26 | Derivation of themes | Were themes identified in advance or derived from the data? | No. inductive coding, page 11 |
| 27 | Software | What software, if applicable, was used to manage the data? | MAXQDA. Page 11 |
| 28 | Participant checking | Did participants provide feedback on the findings? | No. |
| **Reporting** | | | |
| 29 | Quotations presented | Were participant quotations presented to illustrate the themes/findings? Was each quotation identified (e.g. participant number)? | Yes. Page 15-29 and Tables 2-3 |
| 30 | Data and findings consistent | Was there consistency between the data presented and the findings? | Results pages 15-29. |
| 31 | Clarity of major themes | Were major themes clearly presented in the findings? | Tables 2-3 and results |
| 32 | Clarity of minor themes | Is there a description of diverse cases or discussion of minor themes? | Yes, e.g. pages 15 or 17 |

Developed from: Tong, A., Sainsbury, P., & Craig, J. (2007). Consolidated criteria for reporting qualitative research (COREQ): a 32-item checklist for interviews and focus groups. International Journal for Quality in Health Care, 19(6), 349-357. doi: 10.1093/intqhc/mzm042
